# Correction to “Effects of Allogeneic Bone Substitute Configurations on Cell Adhesion Process *In Vitro*”

**DOI:** 10.1111/os.70318

**Published:** 2026-04-13

J. Liu , L. Yang , H. Zhang , J.‐Y. Zhang , and Y.‐C. Hu , “Effects of Allogeneic Bone Substitute Configurations on Cell Adhesion Process *In Vitro*
,” Orthopaedic Surgery 15 (2023): 579–590. 10.1111/os.13395.36453151 PMC9891915

In Figure 6A, the panel for the bone powder group was incorrect:

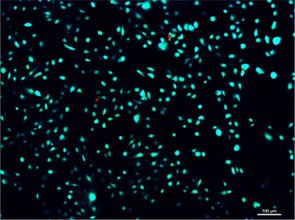

The correct version is shown below:

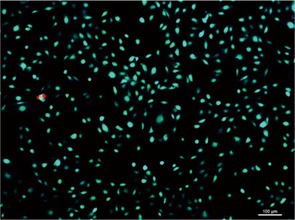

We apologize for this error.

